# Population transcriptomics reveals the effect of gene flow on the evolution of range limits

**DOI:** 10.1038/s41598-022-05248-1

**Published:** 2022-01-25

**Authors:** Katsunori Tamagawa, Kotone Yoshida, Shiori Ohrui, Yuma Takahashi

**Affiliations:** 1 Graduate School of Science, Chiba University, 1-33, Yayoi, Inage, Chiba, 263-8522 Japan; 2 Graduate School of Science and Engineering, Chiba University, 1-33, Yayoi, Inage, Chiba, 263-8522 Japan; 3 Present Address: Graduate School of Life Sciences, Tohoku University, 6-3, Aoba, Aramaki, Sendai, Miyagi 980-8578 Japan

**Keywords:** Evolution, Ecology, Ecological genetics, Population dynamics

## Abstract

One of the most important questions in evolutionary biology is how the spatial distribution of species is limited. Asymmetric gene flow from core populations is suggested to increase the number of poorly adapted immigrants in the populations at the range edge. Genetic load due to migration, i.e., migration load, should prevent adaptation to the local habitat, leading to decreases in distribution range via local extinction or the limiting range expansion. However, few experimental studies have examined the effects of immigration on fitness and natural selection within recipient populations. To investigate the influence of migration load on the evolution of distribution range, we performed field and laboratory observations as well as population transcriptomics for the common river snail, *Semisulcospira reiniana*. This species meets the conditions that migration from source populations can prevent local adaptation in a sink population because they inhabit the broader range of environments, including middle/upper reaches of a river and estuaries within a single river and they may be more vulnerable to being swept away by water currents due to lowered spontaneous (upward) locomotion activity. We found that river steepness was related to the lower distribution limit of *S*. *reiniana*, with a narrower distribution range in the steeper river. Population transcriptomic analysis showed that gene flow was heavily asymmetric from the upstream populations to downstream ones in the steep river, suggesting a greater migration load in the steep river. The number of genes putatively involved in adaptation to the local habitat was lower in the steep river than in the gentle river. Gene expression profiles suggested that individuals achieve better local adaptation in the gentle river. Laboratory experiments suggested that evolutionary differences in salinity tolerance among local populations were only found in the gentle river. Our results consistent with the hypothesis that migration load owing to asymmetric gene flow disturbs local adaptation and restricts the distribution range of river snails.

## Introduction

Traits within a species are known to change gradually across the geographical space. The existence of spatial variation in fitness-related traits indicates that populations can adapt to environmental continuums within the species’ range or within a habitat and that they can adapt to novel environments with inhospitable conditions^1^. In practice, abundant evidence of ongoing adaptation to environmental continuums has been reported^2,3^. Thus, given evolutionary periods of time, any species can adapt to novel environments and extend its range permanently across the globe. However, the distribution of many species ends abruptly, without the presence of obvious ecological or physical barriers, suggesting that adaptation is prevented at the edge of the range^4^. Elucidation of the processes preventing adaptive evolution at range margins is therefore important to our understanding of the evolutionary mechanisms that determine species ranges and the pattern of species diversity on earth.

There are two theoretical explanations for the failure of adaptation at range margins. (i) If migration moves mainly from the central, well-adapted regions of the range into the margins, asymmetric dispersal increases the proportion of poorly adapted immigrants in the margins of a population. The range margins may have lower carrying capacity (i.e., migration load) and consequently, the immigrants fail to adapt to the novel environment. This hypothesis is called the migration load hypothesis^5–8^. (ii) If the range edge is highly fragmented, stochastic events and the low rate of mutational input into marginal populations may limit the availability of locally beneficial alleles, preventing adaptation and therefore range expansion (diversity loss hypothesis)^9^. In general, failure of adaptation along an environmental gradient due to diversity loss is recognized as the predominant process at larger geographic scales because the effect of migration input and migration load is relatively small^10^. In practice, many empirical studies have detected indications that the loss of genetic diversity is the predominant mechanism preventing adaptation. Genetic diversity in a population gradually decreases, and genetic differentiation among the population gradually increases, along the core to edge transects at large geographical scales^11–13^. In contrast, if an environmental gradient is steep at a small spatial scale, the population often receives poorly adapted immigrants from adjacent populations^14^. Thus, the failure of adaptation along the environmental gradient due to migration load is suggested to be the predominant process at a small spatial scale and in species with higher dispersal ability^8,15^. Despite theoretical progress, the actual effect of migration load on the adaptation of populations at range margins remains unclear due to the lack of estimation of gene flow and the genetic and phenotypic signature of maladaptation, although some transplant studies and metanalyses have provided circumstantial evidence for migration load^14,16,17^.

The lotic environment is a suitable system to examine the effects of asymmetric gene flow on local adaptation and range expansion since lotic organisms are exposed to unidirectional water flow and occasional floods. Water currents along a river encourage organisms to move downstream; hence, gene flow occurs most frequently downstream^18–20^. Despite relatively high mobility, the dispersal of fishes is also influenced by stream currents^21–23^. A previous study assessed the phenotypic traits of a stickleback, *Gasterosteus aculeatus*, population inhabiting a pond and its inlet and outlet rivers and showed that the population in the outlet (downstream) of the pond exhibits a phenotype intermediate between that of the river- and pond-adapted fish, whereas the upstream population has a phenotype optimized for the river environment^23^. This inconsistency between the optimal and realized phenotype resulted from frequent immigration from the pond population, following the water flow. Organisms with a limited ability to disperse, such as freshwater snails and other invertebrates, are assumed to be more susceptible to the effects of asymmetric gene flow because passive movements will frequently occur^24–27^. The extent of asymmetric gene flow is also affected by the geographic and physical features of each river since passive movement is caused by environmental factors, such as frequency of floods, nature of the substratum, and the velocity and steepness of the river^18,28^.

The freshwater snail, *Semisulcospira reiniana*, is a common gastropod species in Japan, inhabiting mainly sandy or muddy bottoms of flowing rivers and lakes^29^. This species is broadly distributed within the riverine system including estuaries. Field observation indicated that individuals transplanted into faster currents are prone to migrate downstream^24^. Therefore, comparative research into rivers inhabited by *S. reiniana,* which have different geographic features, is expected to clarify the relationship between gene flow, local adaptation, and the distribution range of species.

Population genetics using single nucleotide polymorphisms (SNPs) is a powerful method used to survey population structures and demography in freshwater environments^26,30,31^. Recent technological developments in next-generation sequencing allow us to analyze both genome-wide SNPs and the transcriptome of wild organisms^14,32–34^. The transcriptomic analysis provides evidence for adaptation and phenotypic change by identifying SNPs located within the mRNA and changes in gene expression^35^. By comparing rivers with steep and gentle gradients, we could infer the impact of the river gradient on local adaptation and the distribution range along the river. Recently, the genetic basis of response to salinity stress in *S. reiniana* was studied by transcriptomic analysis^36^; however, the influence of gene flow on local adaptation and range limit is not known yet in this species. Here we performed population transcriptomics for *S. reiniana* populations collected from rivers of different steepness in order to investigate the influence of gene flow on local adaptation and distribution range. If river steepness influences the direction of migration and local adaptation at range margins, populations in each river should show distinct properties of genetic structure and transcriptional regulation. We also conducted a field survey to examine the influence of river steepness on phenotypic traits. To provide support for the hypothesis that migration load influences the evolution of range limit, the relationship between the river steepness and the lower range limit was analyzed using 12 independent rivers. Our results consistently imply that asymmetric gene flow affects local adaptation and the evolution of range limit.

## Results

### Relationship between river steepness and distribution limits

**Figure 1:**
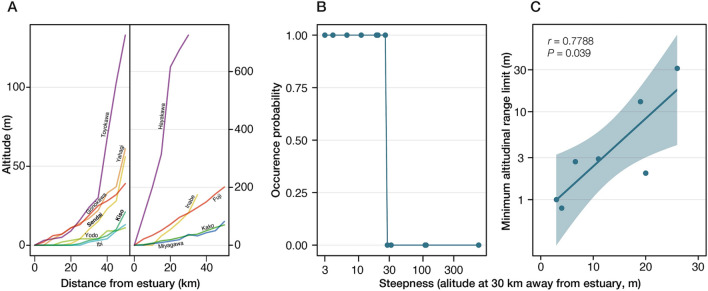
Altitudinal distribution of *Semisulcospira reiniana*. (**A**) Steepness of each river surveyed in this study. Relatively gentle and steep rivers are represented in the left and right panel, respectively. (**B**) Occupancy probability of *S. reiniana* by steepness in each river. (**C**) The relationship between the river steepness and the lower distribution limit of *S. reiniana*. Altitude at 30 km away from estuary is used as a measure of river steepness. Correlation coefficient and *P*-value are shown in the upper left of the panel.

The lower distribution limit of *S. reiniana* was investigated in 12 rivers in Japan—Ibi, Kiso, Yodo, Sendai, Gonokawa, Yahagi, Toyokawa, Miyagawa, Kako, Fuji, Inabe, and Hayakawa Rivers—between 2017 and 2020 (Fig. 1A). The elevation at 30 km from the estuary, a measure of the river steepness, was strongly related to the occurrence of *S. reiniana* in each river (Fig. 1B). In some of the quite steep rivers, no *S. reiniana* were observed up to 50 km from the estuary, while we found that the lower distribution limit was from 10 to 35 km from the estuary in other rivers. The river steepness was significantly correlated with the lower distribution limit of *S. reiniana* (Fig. 1C). The expansion of distribution range into the intertidal zone was observed only in the Kiso River.

### Evaluation of phenotypic adaptation in the peripheral population

**Figure 2:**
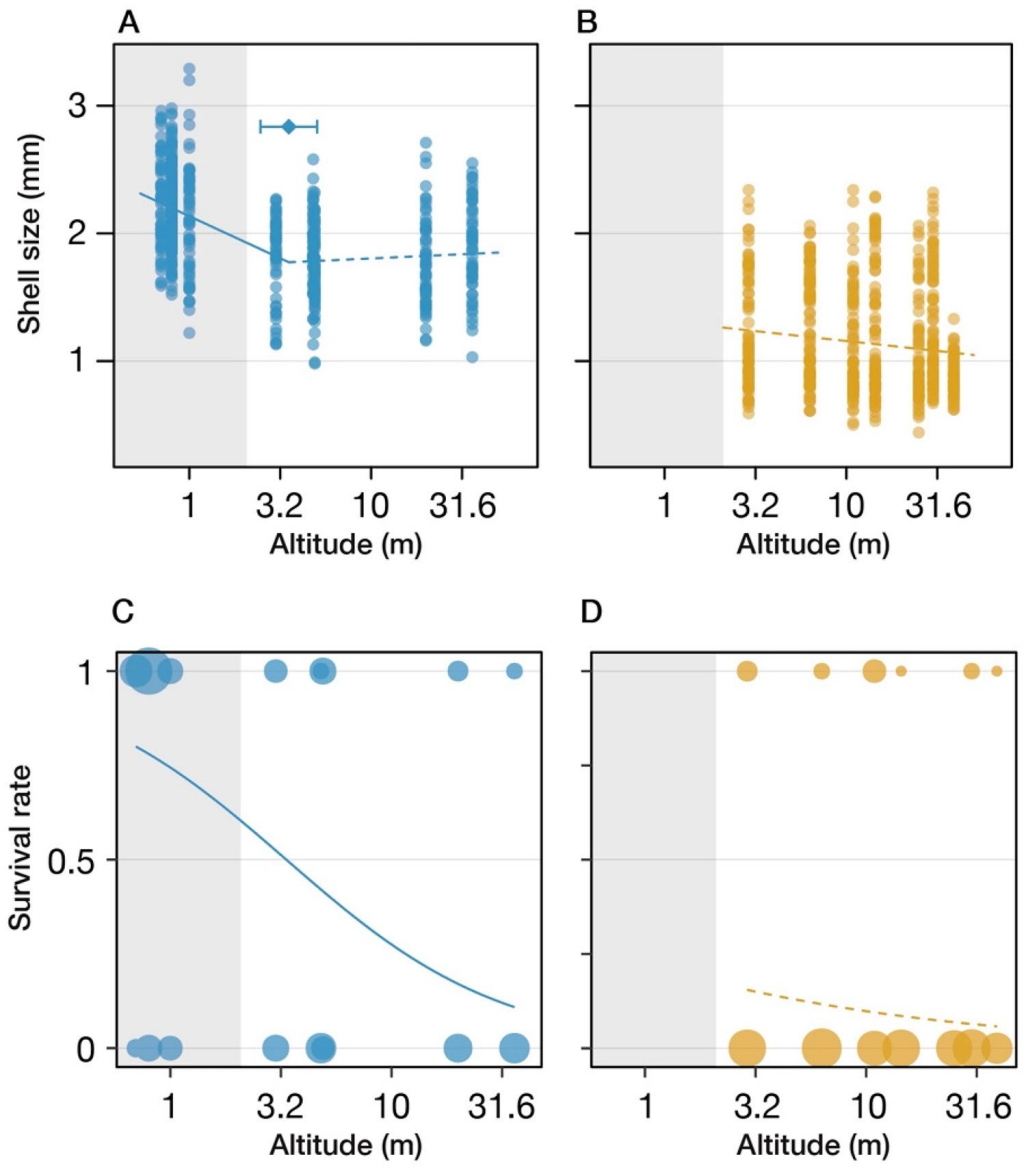
Shell size of juveniles in each population in the gentle (**A**) and steep rivers (**B**). Each line was estimated using a segmented regression method, and the rhombus and whisker represent the significant segmented point and the confidence interval, respectively. The shaded area in each panel indicates the intertidal region in the gentle river (Kiso River). The survival rate of juveniles exposed to 3% saline water in the gentle (**C**) and steep rivers (**D**). The size of each point shows the number of juvenile dead (bottom of each panel) or alive (top of each panel). The curves in each panel were estimated using a generalized linear model, and the line type represents significance.

In the present study, we selected two rivers, the Kiso and Sendai Rivers, with different steepness and enough snails to generate reliable results. In the Sendai River with a relatively steeper slope, the distribution of this species is limited to freshwater habitat, while in the Kiso River with a gentle gradient, the distribution extends into the intertidal zone. The Kiso and Sendai Rivers are referred to as the gentle and steep rivers, respectively. The shell of the juvenile snails was large in the peripheric, downstream population in the gentle river, and a significant switching point for shell size was detected between the brackish water and freshwater populations (Fig. 2A,B). Juvenile snails born from females in each population were subjected to 0%, 1%, 2%, or 3% saline water. Although most of the juveniles exposed to 0% saline water survived, several juveniles died in the water with a higher salinity (altitude, χ^2^ = 23.32, *P* < 0.001; river, χ^2^ = 18.93, *P* < 0.001; concentration, χ^2^ = 574.05, *P* < 0.001; size, χ^2^ = 8.44, *P* = 0.004; altitude × river, χ^2^ = 9.54, *P* = 0.002; altitude × concentration, χ^2^ = 0.26, *P* = 0.61; river × concentration, χ^2^ = 1.48, *P* = 0.22, Fig. S1). The juveniles of downstream populations in the gentle river had higher survival rates than those of upstream populations in water with 3% salinity, whereas most of the juveniles in the steep river died, irrespective of their origins (Fig. 2C,D). A significant effect of body size and altitude of the location of the maternal individuals was detected only in the gentle river (gentle: altitude, χ^2^ = 17.863, *P* < 0.001; shell size, χ^2^ = 15.903, *P* < 0.001, the steep river: altitude, χ^2^ = 1.66, *P* = 0.197; size, χ^2^ = 0.87649, *P* = 0.35), indicating genetic differences in salinity tolerance among populations in the gentle river.

### Reference transcriptome construction and evaluation

Total RNA was extracted from 87 individuals collected from the Kiso and Sendai Rivers. We obtained a total of around 2.5 billion pairs of high-quality reads using Illumina sequencing, and de novo assembly produced 547,731 contigs. After removal of redundancy and screening for protein-coding sequences, 75,670 contigs were retained. We also obtained 184,501 high-quality, full-length Iso-Seq sequences from five individuals, of which 46,891 sequences were retained after removing redundancy. Based on these full-length transcripts, reference-guided assembly was performed to equalize the mapping result among populations, resulting in 18,878 sequences. Reference-guided and de novo assembly were merged to generate 89,850 reftigs with a mean length of 1,425 bp. The reftigs had 96.1% comprehensiveness for the metazoan dataset in Benchmarking Universal Single-Copy Orthologs (BUSCO) v3, and 51,009 sequences had high homology with *C. gigas* protein sequences (E value < 1.0 × 10^−4^). The basic information about the reference construction is summarized in Table S1.

### Population genetic analysis in each river

**Figure 3:**
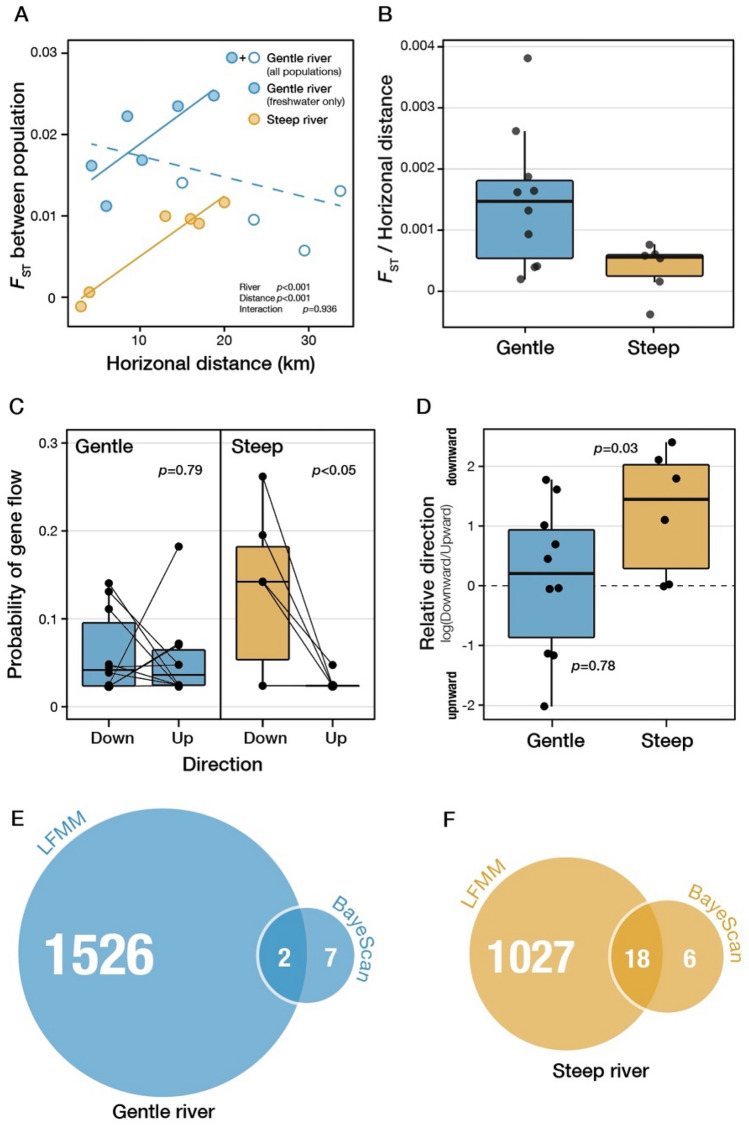
Results of the population genetic analyses. (**A**) The relationship between horizontal distance between populations and the pairwise *F*_ST_ as genetic differences. Filled circles are values between freshwater populations, and open circles are values between fresh and brackish water populations. *P*-values based on generalized linear models are shown in the panel, and the blue dashed line represents the regression using all five populations of the gentle river. (**B**) Ratio of *F*_ST_ to horizontal distance in each river. (**C**) Probability of the downward and upward gene flow between populations in each river. *P*-values based on paired t-tests are shown in each panel. (**D**) The ratio of downward to upward gene flow. Differences between the upward and downward gene flow in each population were tested by one-sample t-tests, and *P*-values are shown beside each box. (**E**), (**F**) The number of outlier SNPs detected by LFMM and BayeScan in each river.

Variant calling and filtering resulted in 7,820 SNPs from the gentle river and 10,721 SNPs from the steep river. Most SNPs were in the UTR, and there were fewer non-synonymous SNPs than synonymous SNPs (Table S2). Using these SNPs, we conducted population genetic analyses (see Table S3 for basic population genetic parameters). To examine the effect of the steepness on the population genetic structure in the two rivers, we analyzed the relationship between geographic distance and pairwise *F*_ST_ among populations. Unlike our expectation, we could not detect the isolation-by-distance (IBD) patterns in gentle river (*r* =  − 0.408, *P* = 0.808) while IBD was detected in steep river (*r* = 0.965, *P* = 0.042, Fig. 3A). However, IBD pattern was also detected in the gentle river when we excluded brackish water populations of the river from the analysis (*r* = 0.795, *P* = 0.042) since all pairwise *F*_ST_ values between a brackish population and freshwater populations were low in the gentle river (Fig. 3A). *F*_ST_ values per geographic distance along a river were significantly higher in the gentle river than the steep river (*P* = 0.002), though such pattern tended to be ambiguous in a case where brackish water population was included (*P* = 0.056). Migration probability estimated using BaysAss3-SNPs indicated that moderate to high gene flow occurs between populations within a river (Fig. S2). Downward migration was significantly stronger than upward migration only in the steep river (Fig. 3C). Asymmetric gene flow, which was assessed by the log-transformed ratio of the downward to upward migration probability, was detected only in the steep river (Fig. 3D). The estimation of genetic population structure was performed using the sparse nonnegative matrix factorization (*snmf)* function implemented in Landscape and Ecological Association (LEA) package of the *R* statistical language. This analysis did not identify different genetic population structures within rivers, and the best number of genetic clusters, *K*, was one (Fig. S3).

The number of outlier SNPs which were strongly associated with elevation (Table S4, S5), was relatively greater in the gentle river (1,528 SNPs) than in the steep river (1,045 SNPs). In the gentle river, 232 SNPs were non-synonymous and involved in a metabolic process, while in the steep river, 170 SNPs were non-synonymous and enriched in Gene Ontology (GO) terms related to response to stimulus and cell development (Table S6, S7). Second, we used *BayeScan,* an *F*_ST_-based method, which detected nine and 24 outlier SNPs in the gentle and steep river populations (Table S8, S9). In the gentle river, two non-synonymous SNPs located in the genes *Bis(5'-nucleosyl)-tetraphosphatase* and *Caskin-1* were detected by both methods; in the steep river, five out of 18 SNPs detected by both methods were non-synonymous (Fig. 3E,F).

### Spatial variation in gene expression in each river

**Figure 4:**
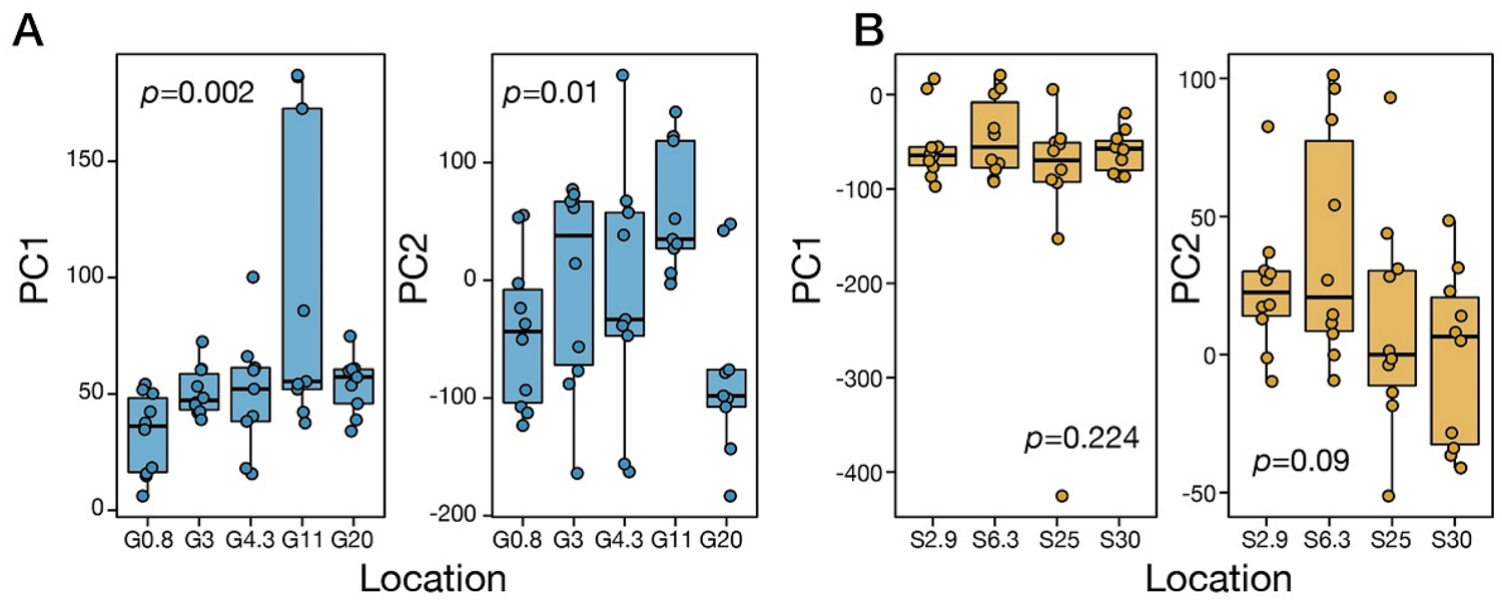
Principal component analysis using expression profiles of both rivers. Boxplots show the variance of PC1 and PC2 values of each population in the gentle (**A**) and steep rivers (**B**). In each panel, *p*-values based on analysis of variance are shown.

Principal component analysis (PCA) based on gene expression profiles identified separation between the rivers, but not among populations within a river (Fig. S4). To clarify the differences in expression profiles among the populations in each river, we extracted and examined the variance of the principal component (PC) values. PC1 and PC2 values differed significantly among populations in the gentle river, but not in the steep river (Fig. 4).

The number of transcripts correlated with an elevation in each river was greater in the gentle river than in the steep river (Fig. S5, Table S10). In the gentle river, 21 transcripts were significantly correlated with elevation (positive: 1, negative: 20), while only five transcripts were correlated with elevation in the steep river (positive: 5, negative: 0).

### Genetic basis of high tolerance to salinity downstream in the gentle river

To detect the genetic basis of tolerance to the highly stressful estuary environment, which includes high salinity and exposure to air during low tide, we conducted an in-depth analysis of the gentle river. The salinity of river water measured by the refractometer was 5, 0, 1, 1, and 1‰ at G0.8, G3, G4.3, G11, and G20, respectively, which show the population at brackish water is exposed to extremely high salinity stress (Table S3). We detected 812 SNPs that were significantly correlated with the salinity in each location, and 147 out of these were non-synonymous (Tables S11, S12). We also identified five genes that were differentially expressed in at least three comparisons between brackish and freshwater populations within the gentle river, and four of the five genes were downregulated in the population living in brackish water (Fig. S6, Table S13). Note that, salinity at tidal (brackish water) area might be underestimated because it was measured at low tide to access the habitat of the species.

## Discussion

In the present study, we demonstrated a relationship between river steepness and the lower distribution limit of a river snail using field data and population transcriptomic data, focusing on SNPs in transcripts and gene expression levels. Although no clear separation of population structures was observed within the rivers, the estimated direction of gene flow and differentiation among the populations supported the assumption that strong downward migration homogenizes the population in the steep river. In the saline tolerance experiment, gene expression analysis and field data suggested that local evolutionary adaptation could be attributed to the expansion of the distribution range to a novel environment, in this case, the intertidal environment, in the gentle river. In contrast, a recent review suggested that most of the studies regarding the effect of gene flow on local adaptation were consistent with the diversity loss hypothesis, and few studies support the migration load hypothesis^37^. Some studies failed to detect any swamping effect on local adaptation under asymmetric gene flow^38^. One possibility is that the swamping effect of asymmetric gene flow is weaker than expected from theoretical studies, owing to complex environmental and ecological conditions, and is hence difficult to demonstrate except in quite simplified systems^17,38^. These results, which appear to contradict ours, might arise from the different consequences of positive and negative gene flow. While the negative effect leads to population collapse at the edge of the range^4,15^, the positive effect of gene flow leads to the formation of stable populations and subsequent range expansion^37,39^. Therefore, populations recognized by us, the abundant and fine population, have probably been subjected to the positive effects of contemporary gene flow, so it is hard to detect the negative effects of gene flow using the existing population. In the present study, we detected the negative effects of gene flow. We overcame this challenge by focusing on whether river snails expand into novel environments and by comparing rivers with different slopes. Our results supported the hypothesis that migration load owing to asymmetric gene flow, along with the flow of the stream, disturbs local adaptation and restricts the distribution range of a river snail. The lack of replication for genetic analysis (we only studied one steep and one gentle river) is a weakness of our study, but the analysis on the relationship between the steepness of river and lower range limit by using multiple rivers can strengthen our hypothesis.

### Genetic differentiation is more apparent in the gentle river

Our results revealed that the population structure within each river is ambiguous, and thus genetic differentiation among the subpopulations is predicted to be small (Fig. S2), probably due to the small geographic distance (less than ~ 35 km) along a river. Previous studies have also suggested that population structure tends to be ambiguous in river snails because they tend to migrate to other locations, mainly downstream, due to water flow, including floods^24–27^. The results of the present study supported the presence of passive asymmetric downward migration and low genetic differentiation among populations in the steep river (Fig. 3C,D). Passive downward migration may predominate over the persistent, aggressive upstream movement in a steep river^40^. In a gentle river, however, aggressive upstream movement contributed to the balance of downstream and upstream migration. Thus, our results suggested that river steepness leads to heavily asymmetric gene flow and introduces poorly adapted individuals or alleles into the downstream population. Field surveys using multiple rivers have identified a significant correlation between river steepness and the range of distribution toward the estuary (Fig. 1B,C). Taken together, in *S. reiniana*, range limitation and local adaptation to novel and harsh habitats probably depends upon the extent of influx from a different environment.

### Detection of natural selection and inference of functional associations within each river

Evidence of local adaptation could be detected in the form of outlier SNPs with different allele frequencies in each population if the population had adapted to the local environment at each elevation, including temperature, sediment, and salinity. In the steep river, functions such as morphogenesis, developmental process, and response to external stimulus were significantly enriched (Table S6), and in the gentle river, GO terms associated with RNA and other metabolic processes were significantly enriched (Table S7). In the present study, however, we found no shared non-synonymous SNPs associated with the elevation in both rivers, and only two GO terms (cellular process and regulation of biological process) detected by LFMM were shared in both rivers. While these findings suggest that local adaptation to the elevation has been accomplished by different genetic pathways in the different rivers, we should be careful to discuss the association between enrichment analysis and the mechanism of local adaptation. To verify the effect of each SNP on local adaptation associated with elevation, we require more evidence of related ecological features in this species and genetic studies for genes where SNPs are located.

In the steep river, 18 SNPs detected by BayeScan were included in 1,045 SNPs detected using LFMM (Tables S4, S8). Many of these SNPs were in the myosin heavy chain (MHC) in the striated muscle and exhibited a clear separation of allele frequency between the upstream and downstream populations. One contig (TRINITY_DN279260_c4_g3_i9) encoded one putative homolog of MHC and contained two non-synonymous alleles in its coding sequence^41^. Typical striated muscle has not been found in “prosobranchs”^42,43^; therefore, MHC could be involved in embryogenesis or early stage muscle development. Since the upstream region has a particularly steep flow and rocky substratum in the steep river, non-synonymous alleles located in the MHC, which are possibly associated with muscle development, were considered to be possible targets of selection. Our pilot analysis revealed that the speed of movement of newly-born individuals whose female parent came from the upstream population was greater than those whose female parent came from the downstream population in the steep river. This observation suggested that only the upstream population, which is a source rather than a sink population, may achieve local adaptation in the steep river (Fig. S7). This pattern is not consistent with the previous studies that have reported a higher dispersal ability at the range edge of populations^44^.

### Differences in transcriptional regulation were greater in the gentle river

In the present study, we considered the expression level of each transcript to be a phenotypic trait and evaluated the state of local adaptation of each river. Since the global gene expression pattern differed significantly with elevation only in the gentle river, individuals inhabiting the gentle river presumably produced the appropriate transcriptional regulation for each local environment (Fig. 4). All individuals were subjected to a uniform environment before RNA fixation, and therefore, observed patterns were assumed to reflect genetic factors rather than phenotypic plasticity.

We observed that more transcripts exhibited expression levels correlated with an elevation in the gentle river than in the steep river (Table S10). These results suggest that local adaptation and habitat expansion rely on transcriptional regulation in *S. reiniana*. Although differences in expression are mainly governed by a mutational change in regulatory regions, in the present study, we focused only on SNPs within the mRNA, and thus we could not demonstrate the existence of SNPs in the regulatory region or investigate these effects on local adaptation. To further investigate the local adaptation in *S. reiniana*, we need to investigate the relationships between these mutational changes and differences in gene expression.

### High salinity tolerance downstream in the gentle river and the genetic basis of local adaptation

In the gentle river, *S. reiniana* inhabits both fresh and brackish water around estuaries, despite the latter being quite a harsh environment, with widely fluctuating tidal levels and daily changes in salinity. The high tolerance to salinity observed in juveniles born from individuals inhabiting brackish water in the gentle river indicated that this population had genetically adapted to the local environment (Fig. 2A,B). While modification of the extracellular matrix, muscle structure, free amino acids, swelling of the cell, de-condensation of perinuclear chromatin, and the induction of stress responses are important functions for osmoregulation and the adaptation of marine mollusks to a hyposaline environment^36,45–54^, adaptation and the responses of freshwater mollusks to intertidal environments has been poorly studied.

Although no clear separation of population genetic structure was found between freshwater and brackish water populations (Fig. S2), 147 non-synonymous SNPs were detected as loci subjected to natural selection associated with salinity in each population (Table S11). These non-synonymous SNPs were enriched in genes associated with the regulation of signaling and response to stimuli (Table S12). They might have arisen during the process of adaptation to large differences in environmental stimuli, such as temperature, hypoxia, sediment, salinity, and tidal rhythm. One non-synonymous SNP was in Adenosine deaminase acting on RNA (*Adar*), which encodes an enzyme that edits adenosine to inosine in pre-mRNA. In *Drosophila melanogaster*, *Adar* is specifically expressed in neurons, and its substrate is primarily mRNAs encoding an array of voltage- and ligand-gated ion channels^55^. RNA editing by *Adar* is hypothesized to fine-tune the neurophysiological properties of animals and mutants and appears to be involved in locomotory behavior, regulation of circadian rhythm, and response to heat and oxidative stress^55,56^. Although the hypothesis that behavioral change based on the non-synonymous mutation contributes to adaptation to the severe environment is reasonable and intriguing, to further discussion, we need investigations including genetic experiments using model organisms.

We detected five genes (GTP-binding protein RAD, Sodium-dependent phosphate transport protein 2A, Heat shock protein 68, Heat shock protein 68 and SAP30-binding protein), which were differentially expressed between brackish and freshwater populations (Table S13). These differences might be the result of adaptation or responses to the intertidal environment, but their function is ambiguous in freshwater invertebrates, including *S. reiniana*. For example, heat shock protein 68 (*hsp68*) maintains homeostasis and prevents cellular degeneration in response to various stressors, and in Pacific oyster, is associated with stress responses to changes in salinity and heat shock^47,50^. The gene expression of *hsp68* increased in response to hyperosmotic, hypoosmotic and heat stress in the Pacific oyster, but our analysis showed lower expression of *hsp68* in populations from brackish environments (Fig. S6). Since all individuals used for the experiment were acclimated in the same conditions before RNA fixation, this result suggests that the difference in expression of *hsp68* between brackish and freshwater populations depends on genetic changes, probably local adaptation, rather than being a response to environmental stress.

In summary, our multifaceted results are consistent with the hypothesis that migration load owing to asymmetric gene flow along the water stream restricts the range expansion of river snails. High tolerance for salinity in downstream population in the Kiso River implies that evolutionary adaptation for local environment might be more easily achieved in the gentle river than in a steep river. To confirm the generality of such a process, we need to conduct similar population transcriptome analyses in several rivers in the future. To reveal the genetic basis of the observed changes in gene expression patterns among populations, we may need to conduct further in-depth analyses of organisms from the rivers.

## Materials and methods

### Relationship between river steepness and distribution limits

The altitudinal distribution range was investigated in 12 rivers in Japan. Field surveys were conducted in nine rivers—the Kiso, Yodo, Sendai, Yahagi, Toyokawa, Miyagawa, Fuji, Inabe, and Hayakawa Rivers—between 2017 and 2020. Sampling sites in each river were selected every 5 km from the estuary to 50 km from the estuary, and sampling of adult snails was carried out for about 30 min at each site. Dense sampling, at sites less than 5 km apart from each other, were conducted around the lower altitudinal limit of the snails’ distributions. For the rivers in which we found at least one individual of the species, the lowest site at which the adults were captured was defined as the lower range limit of the river. For three rivers—Gonokawa River, Ibi River, and Kako River—previously reported occurrence data were used to assess the lower range limits.

Since the downstream areas of large rivers flow through plains in Japan, the difference in steepness between rivers is small at a distance of 0 ~ 20 km from the estuary. In addition, the length of a couple of rivers that we studied is less than 40 km from the estuary. Thus, in the present study, elevations at 30 km horizontally away from the estuary were taken to estimate the “steepness” of each river (Table S14). All statistical analyses in the present study were conducted using R 4.0.0 (https://www.r-project.org/). The relationship between steepness and lower distribution limit in each river was analyzed using Spearman’s correlation calculated using *cor* function.

### Sample collection for laboratory experiments

In the present study, we selected two rivers, the Kiso and Sendai Rivers, which have different steepness and enough individuals to conduct experiments. Though Kiso and Sendai Rivers are relatively gentle in 9 rivers that we studied, Sendai River is one of the steepest rivers in which individuals of *S. reiniana* inhabit at a wide range of elevations. Thus, these rivers are referred to as the gentle river and the steep river, respectively. Adult individuals of *S. reiniana* were collected at the river edges along each river between June and August 2017. For both rivers, since the steepness is similar within 20 km of the estuary, salinity intrusion is caused up to around 20 km from the estuary. The sampling sites were located at a distance of 0–50 km from the estuary, and all sites were around 5 km apart from each other. Individuals were captured by hand. Adults used to examine the tolerance to salinity were collected from 10 and eight locations in the gentle and steep rivers, respectively. Samples used for transcriptomic population genetic analysis came from nine locations, which were 0.8, 3, 4.3, 11 and 20 m above sea level (asl) for the gentle river and 2.9, 6.3, 25, and 30 m asl for the steep river. Sampling locations are named as a combination of the steepness of the rivers (gentle: G, steep: S) and the altitude. For example, the location at 25 m asl in the gentle river and that at 30 m asl in the steep river were referred to as G25 and S30, respectively.

### Saline tolerance experiment and size measurement

To remove environmental effects, saline tolerance was quantified by using newborn snail. Adults captured in the natural populations were maintained in a freshwater aquarium (15 × 20 × 15 cm) in the laboratory at room temperature (~ 25 °C), and juvenile snails (< 5 days old) were collected. The juvenile snails were put into 1.5-ml microtubes filled with 1 ml of 0%, 1%, 2%, or 3% saline water, and stably stored in the dark in the incubator for 5 h. After the 5 h exposure, juvenile snails were transferred to 1.5 ml microtubes filled with 1 ml freshwater to enhance recovering from salinity shock. Since in general a living individual can recover from salinity shock and come out of their shells within an hour after exposure to freshwater, we recorded the viability (dead or alive) and shell size (width) of each individual three hours later after exposure to freshwater. The relationship between the elevation of sampling locations and the survival rates were tested using generalized linear models (GLMs). Shell width was used as the covariate since body size are predicted to influence the survival rate. To identify non-linear changes of shell width, the relationship between the elevation and the shell size was analyzed using segmented regression using the *R* package “segmented” (https://cran.r-project.org/web/packages/segmented/index.html).

### RNA extraction

All individuals from each river were transported to the laboratory, rinsed with freshwater, and kept at constant temperature in an incubator until their dissection around 24 h after rinsing. The columellar muscle tissue was excised and immersed in RNAlater™ Stabilization Solution (preserved at − 80 °C until RNA extraction). Total RNA was extracted using Maxwell® 16 LEV Plant RNA purification kits in accordance with the manufacturer’s instructions. The concentration of each RNA extraction was confirmed using Qubit Fluorometer with Qubit RNA BR Assay kits (Invitrogen, Carlsbad, CA). In the gentle river, 10, 10, 9, 9, and 9 individuals at G0.8, G3, G4.3, G11, and G20 respectively, and in the steep river, 10 individuals at each location were subjected to RNA extraction and RNA-sequencing. Also, individuals for Iso-Seq sequencing using PacBio Sequel system were taken from 0.8 and 1.0 m asl in the gentle river and 2.9, 6.3, and 25 m asl in the steep river to take regionality of transcript repertoire into consideration.

4.5. Reference dataset construction.

For RNA-seq, library preparation and 100 bp paired-end sequencing using the NovaSeq 6000 System (Illumina, San Diego, CA) were outsourced to Filgen (Nagoya, Japan). Sequence read quality was confirmed using *FastQC* (http://www.bioinformatics.babraham.ac.uk/projects/fastqc/), and low-quality reads and bases (Q < 20) were trimmed and discarded using *Trimmomatic*^57^. Filtered short reads were subjected to de novo assembly using *Trinity* v2.6.5 with default parameter^58^. To remove redundancy from de novo assembly contigs, the *CD-hit* program (*cd-hit-est*) was conducted with parameter of sequence identity threshold -c 0.99^59^. Subsequently, *TransDecoder* was conducted to screen protein-coding sequencing, with default parameters^58^.

Iso-Seq was outsourced to the National Institute of Genetics to obtain full-length transcripts. The sequences of each individual were merged using *cd-hit-est* with -c 0.99 and assigned to be reference sequences for reference-guided assembly. Filtered short reads from the RNA-seq were mapped to these reference sequences using *Tophat2* with default parameter^60^, and the reference-guided assembly was constructed from mapped reads using *cuffmerge* and *gffread* implemented in *Cufflinks*^61^. In this study, the contigs that were merged de novo and the reference-guides assembly using *cd-hit-est* with -c 0.99 was defined as “reftigs” for the following analyses. The reftigs were annotated by searching homology with the protein dataset of *Crassostrea gigas* (oyster_v9) using *BLASTX* program. We used only reftig sequences with homology to *C. gigas* proteins for the subsequent analyses (E-value < 10^–4^). Orthology comprehensiveness was assessed using *BUSCO* v3 (https://busco.ezlab.org/).

We identified SNPs in samples from each river in accordance with the best practice for calling variants in RNA-seq of Genome Analysis Tool Kit (*GATK*, https://software.broadinstitute.org/gatk/documentation/article.php?id=3891). For each individual, all reads after quality control were mapped to reftigs using *STAR*^62^, and duplicated reads were identified using *Picard* (http://broadinstitute.github.io/picard). The variant sites were detected using HaplotypeCaller with minimum confidence threshold of 20 and merged for each river using GenotypeGVCFs^63^. Then, variants were filtered using VariantFilteration with threshold of FisherStrand > 30 and QualByDepth < 2. Loci that had alleles observed in at least 5% of individuals existed in all individuals within each river, or were bi-allelic, were retained using *VCFtools*^64^. To estimate the impact of each SNP on phenotypes, the SNPs were classified according to their positions in the mRNA: synonymous, non-synonymous, or UTR, using *snpEff*^65^. Classification was conducted according to the GFF file output from *TransDecoder*.

### Population genetic analysis

To avoid the effect of artifacts or sequencing error, deviation from the Hardy–Weinberg equilibrium were calculated using the—hwe option implemented in *VCFtools* and were excluded from the dataset (*q* < 0.05). Linkage disequilibrium was calculated using *Plink* v1.9^66^ and one member of each tightly linked SNP pair was excluded from subsequent analyses (*r*^2^ > 0.8). The pairwise *F*_ST_ between populations within each river, mean of the allele number, and the observed and expected heterozygosity were calculated using *Arlequin*^67^. The existence of isolation-by-distance (IBD) was tested using the pairwise *F*_ST_ by using *mantel* function in vegan package (https://cran.r-project.org/web/packages/vegan/index.html). The extent of IBD within a river would reflect the migration rate of *S. reiniana* and the connectivity between populations, and thus we were able to infer differences in gene flow between rivers. Also, the degree of IBD between rivers was compared using GLM. In this analysis, we used only freshwater populations to compare IBD between the rivers. *F*_ST_ values per horizontal distance along a river were compared between rivers using a Wilcoxon rank sum test in exactRankTests package (https://cran.r-project.org/web/packages/exactRankTests/index.html). Gene flow within each river was estimated using a Bayesian method implemented in *BA3-SNPs*^68,69^. Using this method, we were able to estimate the extent and direction of gene flow in the past few generations using a large SNP dataset. We estimated gene flow using the Bayesian estimation and sampled every 1,000th iteration from 10,000,000 iterations after a burn-in of 1,000,000 steps. Asymmetric migration was tested using one-sample t-tests for the ratio of downward to upward estimated migrations between locations within each river. For the assignment test, we used the sNMF function implemented in LEA package^70^ in R, which is fast and efficient compared with conventional methods^71^. The population structure of each river was estimated using sNMF with 50 repetitions, using neutral loci, after removing loci that deviated from HWE and linkage disequilibrium. In the gentle river, there were 4,544 SNPs, and in the steep river, there were 6,142 SNPs).

Loci subjected to natural selection were detected using two different methods using loci detected in at least 90% of individuals within each river (gentle: 25,134 SNPs, steep: 24,665 SNPs). First, LFMM function implemented in *LEA* package were used to screen SNPs whose frequencies correlated with elevation. The number of latent factors was determined using the genetic structure identified by the sNMF function in *LEA* package. We ran LFMM for 10,000 iterations with 5,000 burn-in iterations and regarded SNPs that had a false discovery rate (FDR) of < 0.05 as calculated by the *p.adjust* function in R as outliers associated with elevation. Second, *F*_ST_ outlier tests was conducted using a Bayesian method with *BayeScan* 2.1^72^. *BayeScan* with a prior odds value of 10 was run for 5,000 iterations with 500,000 burn-in and identified SNPs that had an FDR of < 0.05. We detected genes related to habitat expansion into brackish water in the Kiso River using LFMM and the salinity measured by refractometer at each elevation. GO terms were linked to each reftig using homology with the top-hit to a *Drosophila melanogaster* protein dataset (FlyBase r6.36, *BLASTP* E-value < 10^–4^), and GO enrichment analysis was performed using Panther^73^.

### Expression analysis

To examine the degree of local adaptation produced by transcriptional regulation, we quantified the expression levels of the transcripts in each individual and investigated the differences within and between the river under constant conditions. The transcript abundance in reftigs was quantified using RSEM^74^ with bowtie2^75^ as the alignment program. First, PCA based on fragments per kilobase of exon per million mapped reads (FPKM) values of all reftigs that has homology with *C. gigas* protein dataset was conducted using the *prcomp* function in R, and the pattern of gene expression (PC values) within rivers was analyzed by ANOVA using car package in R. Genes responsible for the habitat expansion into brackish water were explored by comparing expression levels between brackish water and freshwater populations in the gentle river. We tested the differences of expression levels after trimmed mean M-value (TMM) normalization between brackish water and freshwater using edger and TCC package with FDR of < 0.05^76,77^. To investigate the relationships of the local environment in the population and adaptation with transcriptional regulation, we estimated Pearson’s correlation between the elevation and TMM normalized expression values of each transcript within each river.

## Supplementary Information

Supplementary Information 1.

Supplementary Information 2.
