# Supplementary material for: Population transcriptomics reveals the effect of gene flow on the evolution of range limits
Source: Sci Rep. 2022 Jan 25;12:1318. doi: 10.1038/s41598-022-05248-1 (PMC8789792; doi:10.1038/s41598-022-05248-1)
Supplement: Supplementary file 1 — Supplementary Information 1. [file 41598_2022_5248_MOESM1_ESM.docx]

**Supplemental Figures**


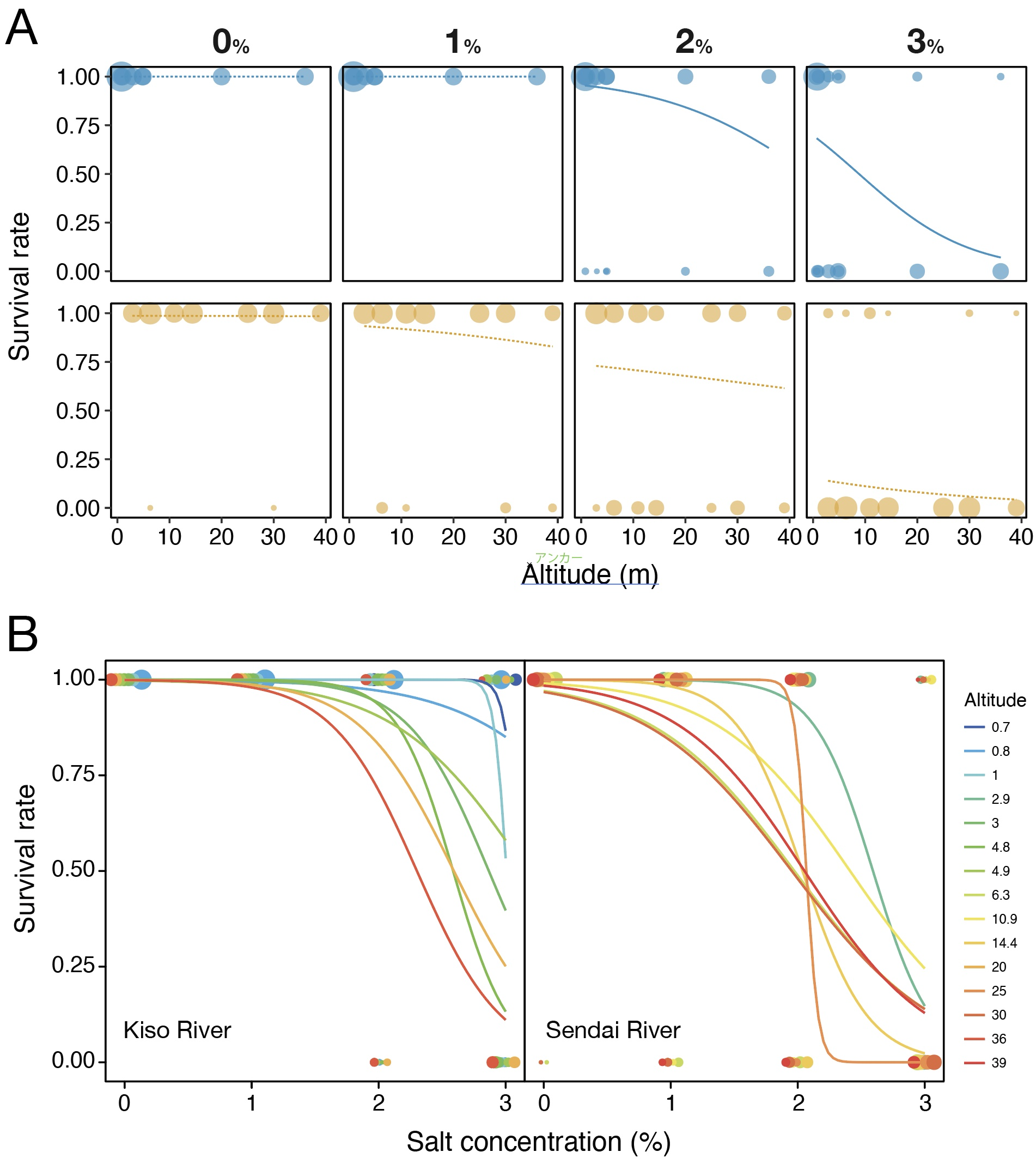


# Figure S1. Survival rate of the juvenile population exposed to 0%, 1%, 2%, or 3% saline water. (A) Changes in survival rate along altitude for gentle (upper panels) and steep river (lower panels). The size of each point represents sample size. The curves in each panel were estimated using the generalized linear model, and the line type represents significance (solid line: P < 0.05, dotted line: P ≥ 0.05). (B) Decline in survival rate with increasing salt concentration for each population.

**
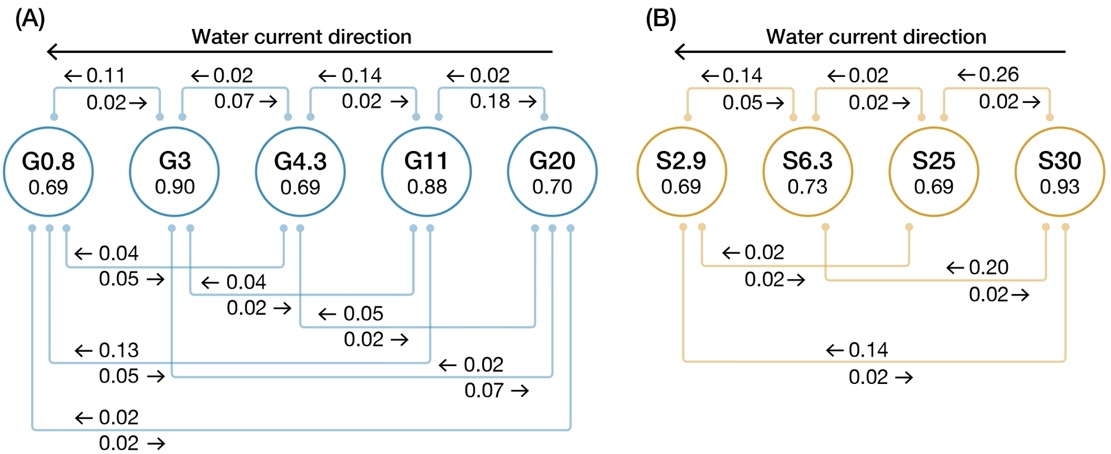
**

**Figure S2.** The extent and direction of gene flow between populations (Circle) estimated using BayesAss in gentle (A) and steep (B) river. The values above and below each line represent the probability that an individual in a given population migrated from other populations in the past few generations. Meanwhile, values in each circle represent the probability that individual derived from this population. The arrows indicate the direction of the gene flow.

**
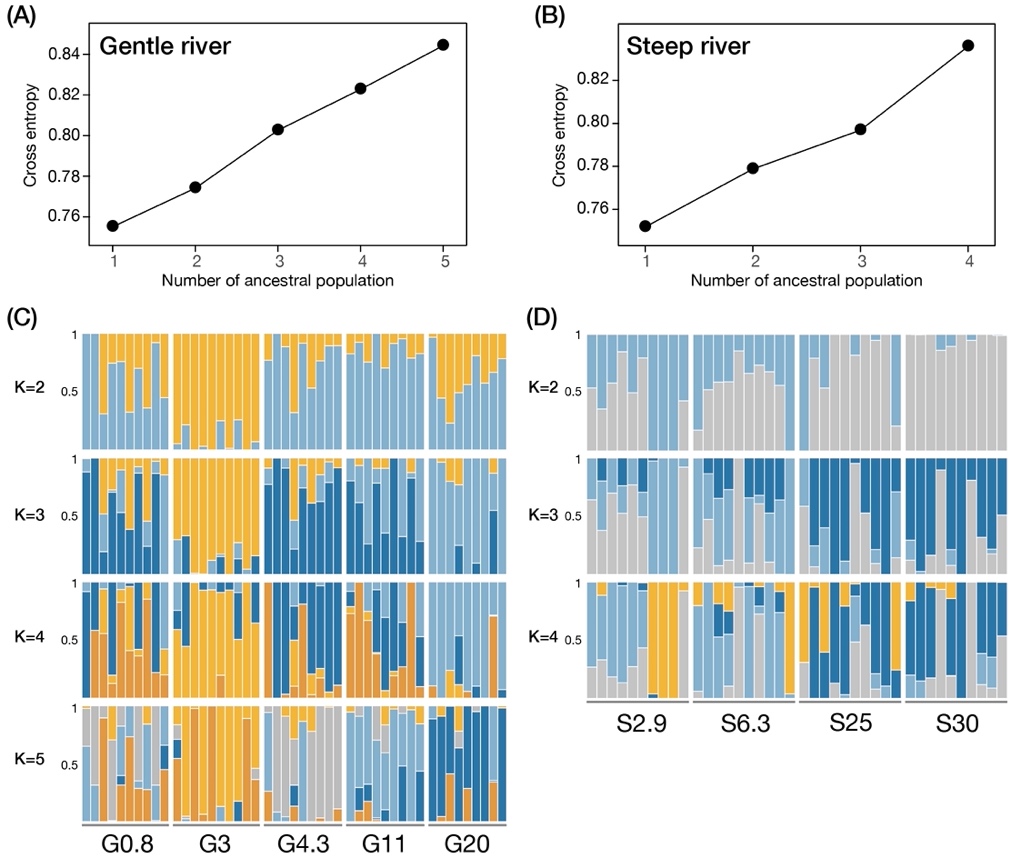
**

**Figure S3.** Population structure estimated using sNMF using only neutral SNPs in the gentle (left) and steep (right) river. The upper plots show the cross entropy for the number of ancestral populations. The bar plot below each plot represents the probability that an individual originates from each ancestral population when a given number of ancestral populations are provided (K represents the number provided for each analysis).

**
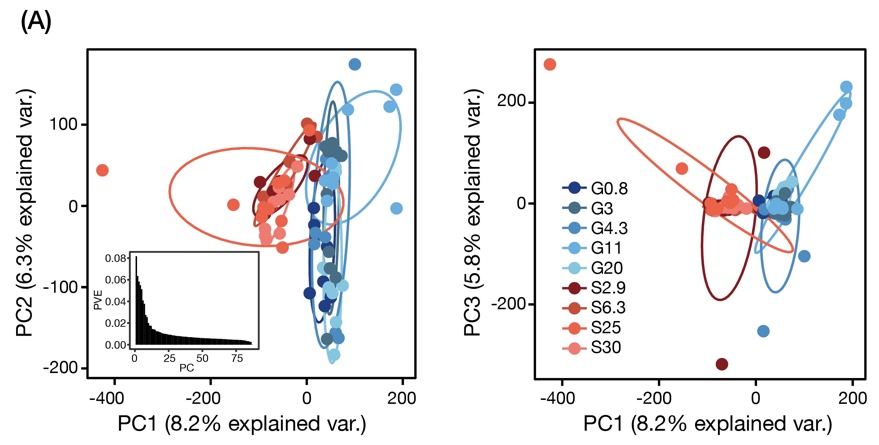
**

**Figure S4.** Principal component analysis (PCA) using expression profiles of both rivers. PCA plot using PC1 and PC2 in the left panel and PC1 and PC3 in the right panel are shown. Scree plot of this PCA is also shown in the left panel, where the y-axis is the proportion of variance explained (PVE). The colors listed in the right panel represent the populations.

**
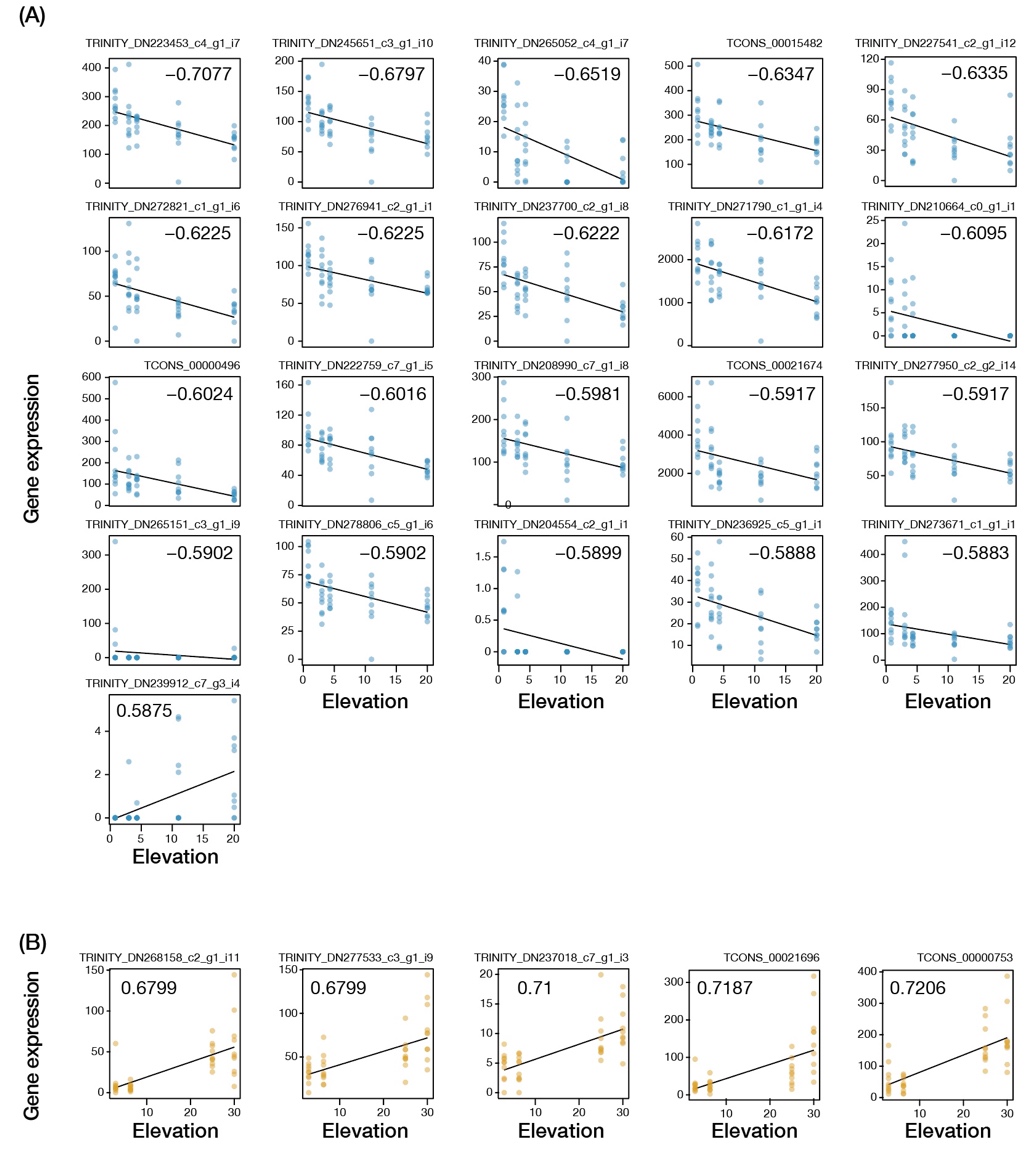
**

**Figure S5.** Genes whose expression values are correlated with the elevation of each population in gentle (A) and steep (B) river. Spearman’s correlation coefficients are represented in each panel.


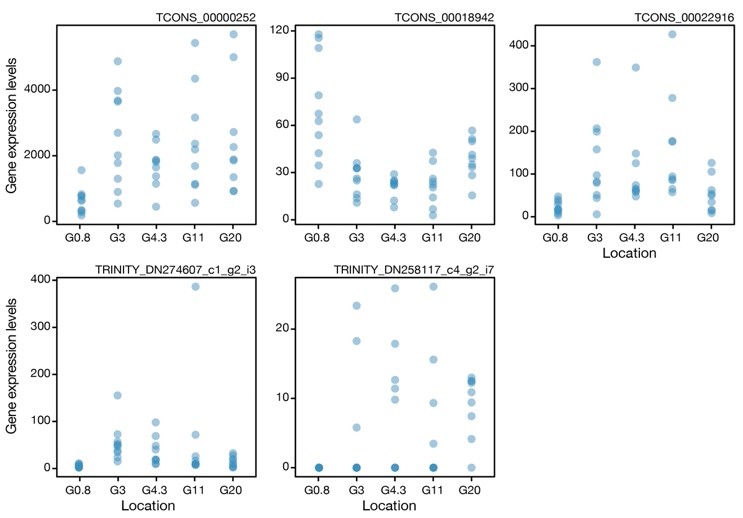


**Figure S6.** Differentially expressed genes in at least three comparisons between the brackish and freshwater population in the gentle river.

**
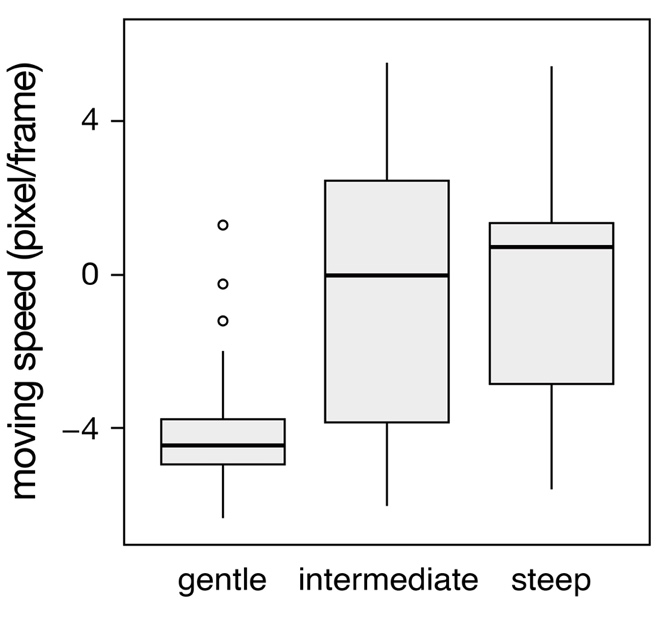
**

**Figure S7.** Altitudinal variation in the moving speed of juvenile snails in Sendai river. The movement distance of juvenile snails from the laboratory was quantified using a 10-min movie. The snails moved freely on a small dish (φ 20 mm). To remove the effect of body size on moving speed, residual from a regression of shell size on moving speed was analyzed. Juveniles whose parent was derived from a higher altitudinal population (steep) moved more quickly than those from the lower altitudinal population (gentle) (χ^2^ = 18.8, *P* < 0.001).
